# Delivery of a Healthy Child Through International Gestational Surrogacy 10 Years Following Female Fertility Preservation and In Vitro Fertilization (IVF) for Recurrent Breast Cancer: A Case Report

**DOI:** 10.7759/cureus.43827

**Published:** 2023-08-20

**Authors:** Elias Tsakos, Emmanouil M Xydias, Apostolos C Ziogas, Nikolaos Tsagias, Konstantina Pappa, Afroditi Stergioula, Kanelina Bimpa

**Affiliations:** 1 Obstetrics and Gynaecology, EmbryoClinic IVF, Thessaloniki, GRC; 2 Medicine, School of Health Sciences, University of Thessaly, Larissa, GRC; 3 Embryology, EmbryoClinic IVF, Thessaloniki, GRC; 4 Legal Department, EmbryoClinic IVF, Thessaloniki, GRC; 5 Breast Surgery, EmbryoClinic IVF, Thessaloniki, GRC

**Keywords:** frozen-thawed embryo transfer (fet), slow-freezing, embryo cryo-preservation, fertility legislation, fertility preservation, recurrent breast neoplasms, cross-border reproductive care, gestational surrogacy

## Abstract

Assisted reproduction technology (ART) has made considerable progress in recent years; in particular with regard to cryopreservation, long-term storage, successful thawing, and embryo transfer of cryopreserved embryos. Regarding gestational surrogacy, progress has been made in the areas of awareness, social acceptance, regulation, legislation, availability, streamlining, and optimization of cross-border care. The above is being highlighted in the current presentation of a particularly challenging and novel case. A 43-year-old woman visited our clinic in Greece, seeking international gestational surrogacy due to recurrent breast cancer which rendered her medically unfit for pregnancy. Ten years before her initial visit to our clinic the patient had undergone fertility preservation due to breast cancer, her oocytes had been fertilized with her husband’s sperm, and the embryos were cryopreserved and stored in a fertility clinic based in the United Kingdom. The stored embryos were transported to Greece, thawed, and successfully implanted to the selected gestational surrogate. Following an uneventful pregnancy, the surrogate delivered a healthy girl. This successful outcome exemplified innovation, motivation, and hope and may represent a paradigm of team scientific excellence associated with positive patient outcomes. Furthermore, this case constitutes the successful culmination of major advances made in various different sectors of cross-border reproductive care; laboratory, clinical, legal, ethical, and logistical.

## Introduction

Infertility is becoming an increasingly prevalent condition in the modern world, with increasingly more couples and single individuals seeking assisted reproduction technology (ART) services [1]. Given the diverse causes of infertility and the potential contribution of both partners to it, a plethora of ART services are available, tailored to the needs of each individual case. Fertility preservation is one such service, which may be applied prior to surgical or medical treatment that may adversely affect the fertility potential of women and men.

This process entails the extraction, cryopreservation, and storage of genetic material or embryos and its use at a later time when it is medically safe and indicated or based on the couple’s family planning. In view of the above, long-term cryo-storage of embryos has been a fairly common occurrence.

However, in certain challenging cases, even if fertility preservation has been successfully performed, pregnancy and delivery may not be medically indicated or feasible, due to a plethora of different reasons. Such reasons may include anatomical conditions (absence of uterus, severe uterine abnormalities, either congenital or acquired), medical reasons (severe thrombophilia, antiphospholipid syndrome, severe renal insufficiency, etc.), iatrogenic (hysterectomy, chemotherapy, etc.) and many more. For such cases, another ART technique, gestational surrogacy (GS) may be indicated, as an advanced, final-line fertility treatment option. Entailing the implantation and gestation of an embryo created from the genetic material of the intended parents to the uterus of a surrogate mother, GS is a challenging, but immensely rewarding and effective program and, sometimes, the only option available to certain individuals to become parents.

The aforementioned ART techniques may be further complicated in cases of cross-border patients and when genetic material is required to be transported between different fertility clinics based in foreign countries. Under these circumstances, legal and logistical considerations further exacerbate the already difficult journey of the patients [2]. Despite all these challenges, the constant evolution of ART services, equipment, protocols, legislation, and logistics has led to remarkable progress in modern fertility care and improved patient outcomes.

The aim of this report is to highlight this remarkable progress through the presentation of a case of successful delivery of a healthy child through cross-border GS with the use of cryopreserved embryos stored for over a decade abroad, following fertility preservation for breast cancer.

## Case presentation

The current report presents the case of a 43-year-old woman and her 48-year-old husband who became parents via an international gestational surrogacy programme. The couple first visited our clinic in March 2019. The woman had been diagnosed with breast cancer in 2010, which was initially treated surgically and subsequently with adjuvant chemotherapy, hormonal therapy, and radiotherapy. Prior to her breast cancer treatment, the patient had undergone fertility preservation in the UK at the age of 34 years.

Fertility preservation and cancer treatment

Following controlled hormonal ovarian stimulation, oocyte retrieval, in vitro fertilization (IVF), and intra-cytoplasmic sperm injection (ICSI) with the husband’s sperm, five embryos were created. The embryos were cultured using the SAGE® sequential medium, until they reached the blastocyst stage on the fifth day of embryo culture, with one embryo reaching the blastocyst stage on the sixth day of culture. Subsequently, the embryos were cryopreserved via the slow-freeze method, using the Quinn’s Advantage® blastocyst freeze kit (Ref. No: ART-8015). The embryos were stored in PETG® 91mm embryo straws (Ref. No: 006433) and remained in storage at the UK-based fertility clinic.

The patient continued breast cancer treatment with tamoxifen for five years post-operatively and adhered to a frequent follow-up protocol. Two years following the discontinuation of her hormonal treatment, the patient initiated fertility investigations in the UK with a view to proceed with IVF treatment by means of frozen/thawed embryo transfer. However, three years after the discontinuation of her hormonal treatment (eight years after the initial breast cancer diagnosis and treatment), the patient suffered from breast cancer recurrence and she underwent bilateral mastectomy and bilateral axillary lymphadenectomy. Histopathological analysis revealed a G2 invasive ductal adenocarcinoma in the left breast, 17mm in diameter, without lymph-node metastases, positive for estrogen receptors (ER-positive) and negative for human epidermal growth factor receptor 2 (HER2-negative). Postoperatively, the patient was treated with letrozole and goserelin.

Gestational surrogacy decision and preparation

Given the patient’s medical history, IVF and pregnancy were contraindicated and gestational surrogacy was offered to the couple as a fertility option. Due to availability issues in the UK, the couple opted for international gestational surrogacy care in our clinic in Greece. The cryopreserved embryos were subsequently transported to our clinic, with the use of a specialized biological courier service, certified for cross-border transport of human genetic material. Following the appropriate medical screening of the gestational surrogate, in addition to several other logistic and legal preparations, including the issuance of a parental court order, she was ready to proceed with the embryo transfer.

Embryo transfers and pregnancy outcomes

The gestational surrogate underwent an embryo transfer on 20/10/2020. One embryo was thawed, using the FertiPro® Warming Kit (Lot No: FP20FVW10) and cultured for four hours in a continuous single culture, supplemented with human serum albumin; however, the embryo did not progress satisfactorily and was hence discarded. Two embryos were thence thawed, with the use of the same protocol and, following laser-assisted hatching (LAH), progressed normally and were subsequently transferred to the gestational surrogate. The embryo transfer was performed with the use of a Wallace Classic® embryo transfer catheter (CooperSurgical®). Under transabdominal ultrasonographic guidance, the catheter was gently passed through the cervix and into the endometrial cavity until the catheter’s tip reached approximately the second third of the endometrium, just above the level of the isthmus. Subsequently, the embryos were gently injected into the endometrium, and their position was verified via ultrasound.

**Figure 1:**
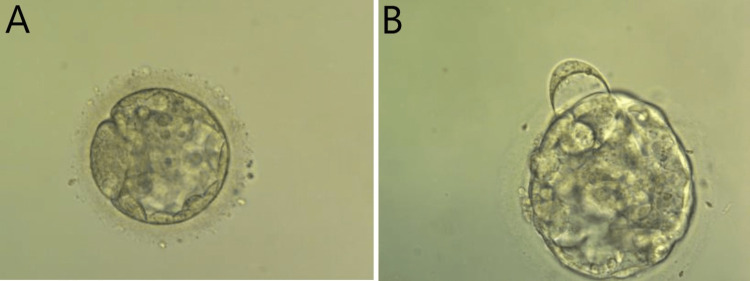
Microscopy images of thawed embryos prior to the first embryo transfer. A: Transfered blastocyst graded as 3BB quality; B: Transfered blastocyst graded as 3AB quality.

**Figure 2:**
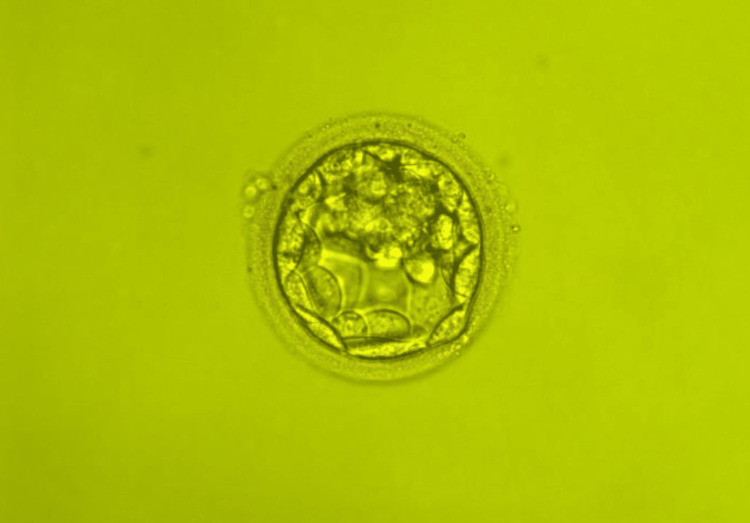
Microscopy image of the thawed embryo prior to the second embryo transfer. Transfered blastocyst graded as 2AA quality.

The transferred blastocysts were of 3BB and 3AB quality according to the Gardner score scale [3] (Figure 1). This embryo transfer resulted in a single pregnancy and a complete miscarriage at six weeks gestation. A second embryo transfer with the same gestational surrogate was performed on 18/3/2021. A single embryo was thawed using the FertiPro® Warming Kit (Lot No: FP20FVW10), cultured for three hours and 30 minutes in a continuous culture supplemented with 20% human serum albumin, and transferred into the surrogate’s uterus with the same routine technique as described previously. The transferred blastocyst was 2AA quality (Figure 2). Embryo characteristics, embryo transfer parameters, and outcomes are summarized in Table 1.

**Table 1:** Summary of the received embryo characteristics and embryo transfer parameters. *Embryo quality grades according to the scale by Gardner et al. [3] STR: straw, ICSI: intra-cytoplasmic sperm injection, IVF: in vitro fertilization, LAH: laser assisted hatching, ET: embryo transfer

Storage straw No	Freeze date	Embryo growth state	Embryo Quality at freezing*	Fertilization method	Thaw date	LAH	Embryo Quality at thaw*	Thaw outcome	ET outcome
STR 1	9/11/2010	Day 5	2BC	ICSI	10/20/2020	Yes	-	Poor embryo development	No ET
STR 2	9/11/2010	Day 5	1BC	ICSI	10/20/2020	Yes	3AB	ET	Miscarriage at 6 weeks
STR 3	9/11/2010	Day 5	3CC	IVF	10/20/2020	Yes	3BB	ET	Miscarriage at 6 weeks
STR 4	9/11/2010	Day 5	2BC	IVF	03/18/2021	Yes	2AA	ET	Pregnancy & delivery
STR 5	9/11/2010	Day 6	4CC	IVF	-	-	-	-	Remains in cryo-storage

This second embryo transfer was successful, resulting in a healthy singleton and uneventful pregnancy, which resulted in a spontaneous vaginal delivery of a healthy girl at 36 weeks and three days gestation. The neonate weighed 2620 g, had normal appearance, pulse, grimace, activity, and respiration (APGAR) scores, and required no specialized interventions and intensive care. At present, at 20 months old, the child has demonstrated normal physical and cognitive development, with no abnormalities whatsoever.

## Discussion

The present case report highlights the significant, rapid advances of ART services in recent years, in both laboratory, clinical, and logistical terms. Firstly, the successful, long-term cryopreservation of genetic material collected prior to cancer treatment and its successful use a decade later. Secondly, the development, expansion, and availability of gestational surrogacy as an option for women to whom pregnancy is contraindicated. Thirdly, the international aspect of fertility care, with legislation and services that facilitate direct communication and coordination between clinics in different countries, forgoing lengthy and complicated bureaucratic procedures for cross-border transfer of genetic material and the increased popularity of “fertility cross border care”.

The evolution of fertility preservation options has considerably affected the landscape of reproductive medicine. In this case, the patient underwent fertility preservation prior to her treatment for breast cancer. Given the fact that breast cancer is the most frequent malignancy in females, with over 2.2 million new cases recorded worldwide in 2020 alone [4], many women seek similar fertility preservation solutions. Additionally, since adjuvant hormonal treatment for breast cancer may be extended up to five or even 10 years [5], long-term effectiveness and safety of embryo preservation are of paramount importance in order to ensure optimal fertility outcomes. Embryo cryopreservation technology has advanced rapidly since its initial development in the 1970s, leading to the modern vitrification methods which have been widely established in the present times, offering a wide variety of options to the modern fertility specialist [6,7]. This evolution and optimization of laboratory equipment, skills, and protocols have led to the utmost safety of long-term cryo-storage, thawing, and transferring of preserved embryos. Shi et al. [8] successfully demonstrated that the duration of cryo-preservation of slow-frozen embryos had no statistically significant impact on survival, implantation, pregnancy, and live birth rates, even when comparing embryos preserved for six to 12 months to those preserved for over 84 months. Similar observations were made by Liu et al. [9], who concluded that storage time had no significant effect on survival, damage, implantation, pregnancy, live birth, or multiple pregnancy rates and thus had no negative influence on further development. Our observations in the current case are in complete agreement with the aforementioned conclusions and are indicative of the progress made in the technical aspects of fertility preservation.

Apart from the improvements made in the laboratory aspect of ART, several advances have been made in the clinical and legal aspects as well. Gestational surrogacy (GS) entails the gestation by a surrogate carrier, usually a young, healthy, and motivated woman. GS is typically reserved as an advanced option for select cases with specific indications, such as in this case, and has become popular recently. Data from the USA indicate that GS was representing 1% of all ART procedures in 1999 and 2.5% in 2013 [10,11], with this upward trend being likely to continue. While data is still lacking, due to the small number of available cases, a study by Attawet et al. [12] that included a total of 81 gestational surrogates with 170 frozen embryo transfers, demonstrated a cumulative live birth rate of 50.6%. The majority of gestational surrogates, similar to our case, underwent frozen embryo transfer and delivered vaginally, however, no data is provided on the duration of embryo cryopreservation prior to embryo transfer. Peters et al. [11] included 63 gestational surrogates in their study and demonstrated a live birth rate of 55.5%, however, these results are from synchronized cycles with fresh embryo transfers in addition to frozen ones. Overall, GS was regarded as an effective program for women with anatomical or other pathology that constituted a contraindication for pregnancy [11,12].

Despite the recent rise of GS in popularity, there are some ethical considerations that are still raised [13]. One of the most prevalent and the most highlighted is the presumed exploitation of surrogates since many argue that poor and vulnerable women are primarily attracted to such programs and that GS reinforces historical and cultural gender stereotypes about female decision-making and biological destiny [13]. An additional concern has been the well-being of children born through GS, since many fear that said children may be viewed as objects, products of a financial transaction [13]. However, medical research has shown that in countries where legislation is in place to regulate surrogacy, there is no evidence of either surrogate exploitation or child endangerment, with studies showing that children born through GS may be even better adjusted than their traditionally conceived counterparts [13]. Nevertheless, several national and supranational organizations have made and are still making attempts to improve the legal and ethical standards of GS [13], instilling hope in many intended parents worldwide.

Despite the rising popularity of GS and all the above considerations, legislation is not yet in place in every country to regulate its use in clinical practice; with added legal issues regarding child custody being also a consideration in many contexts [13]. Greek law permits and regulates GS for cases where it is medically indicated, with a formal application along with the necessary medical documentation being a prerequisite for consideration. An added advantage of this legislative process in Greece is the issuance of the court order for GS prior to the initiation of the ART procedures and well before delivery. This arrangement ensures legal safety for the unborn child (or children), as custody is certified a priori, and acts as an assurance for the intended parents and gestational surrogate alike, since it minimizes unexpected legal complications during pregnancy and after the delivery.

The existence of such legislation regarding GS, in particular with regard to the legal safety of the intended parents and gestational surrogate alike, is indicative of the astonishing progress made in reproductive medicine in recent years and is a significant contributor to the increase in cross border fertility care observed in Greece and other countries where similar legislation applies [14]. This is further facilitated by the constant streamlining of communication and coordination between fertility clinics in different countries and sperm and/or oocyte banks and specialized genetic material courier services which ensure safe and legal cross-border transfer of genetic material [14]. These significant improvements on the logistical side of things have greatly increased the availability of fertility services and have allowed for the advancement of fertility care to a truly international level. This contribution, which may be taken for granted by many, was particularly highlighted in the management of the present case, as it happened during the COVID-19 pandemic. As such careful communication and coordination between clinics, courier services, and the couple was required due to travel and clinic operation restrictions that applied in different countries at different times and in order to ensure the protection and safety of all parties involved from COVID-19 infection [15,16].

## Conclusions

This report presented the case of the delivery of a healthy child via GS from cryopreserved embryos, which were created a decade ago in the context of fertility preservation due to breast cancer diagnosis and treatment. This case highlights a culmination of many notable advances in the field of ART throughout the years, such as the safety and success of long-term embryo cryo-preservation, the expansion of altruistic GS programs, and the cross-border interconnection of fertility clinics worldwide. As ART technology and scientific protocols continue to evolve, more similar successful cases are likely to emerge and eventually become the norm. In addition to technological evolution and scientific advancement, the continued collaboration between medical practitioners, lawmakers, and ethicists is vital in order to ensure similar improvement in the ethical and legal aspects of ART services. It is to those ends that research, development, legislation, and discussion in the field of ART should strive towards, constantly optimizing the medical aspect while ensuring that the ethical and legal aspects are aligned with the evolving medical landscape; with the ultimate objective of assisting individuals in their journey to parenthood.
